# Molecular Background of the Lychee Aroma of *Vitis vinifera* L. ‘Muscaris’

**DOI:** 10.1021/acs.jafc.3c08298

**Published:** 2024-01-12

**Authors:** Xingjie Wang, Stephanie Frank, Martin Steinhaus

**Affiliations:** † Technical University of Munich, TUM School of Natural Sciences, Department of Chemistry, Lichtenbergstraße 4, 85748 Garching, Germany; ‡ Leibniz Institute for Food Systems Biology at the Technical University of Munich (Leibniz-LSB@TUM), Lise-Meitner-Straße 34, 85354 Freising, Germany

**Keywords:** Muscaris, Muskateller, Vitis vinifera L., lychee aroma, (2*S*,4*R*)-rose oxide, geraniol, aroma extract dilution analysis (AEDA), stable isotopically substituted odorant, odor activity value (OAV), odor reconstitution

## Abstract

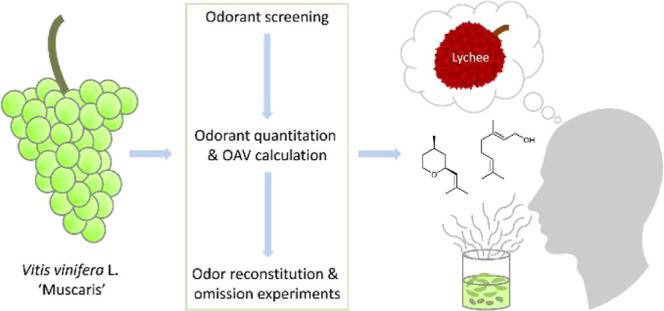

Muscaris is a modern white grape variety with good fungal resistance and a pleasant aroma, the molecular background of which was unknown. A comparative aroma extract dilution analysis applied to Muscaris grapes and grapes of the father variety Muskateller revealed little differences and resulted in 39 and 35 odorants, respectively. Sixteen odorants exceeded their odor threshold concentrations. Odor reconstitution and omission experiments showed that the distinct lychee note in the aroma of the Muscaris grapes was generated by the combination of (2*S*,4*R*)-rose oxide and geraniol. This finding will guide further molecular research on the transfer of the lychee note into wine and may also be helpful for the targeted breeding of new grape varieties.

## Introduction

Muscaris is a relatively new *Vitis vinifera* variety with an intense aroma and good resistance to downy mildew, powdery mildew, and botrytis. Muscaris is early ripening. The berries remain green, even at high must weights. It is particularly suitable for producing dessert wine and dry wine.^1^ The cultivation area of Muscaris increased in recent years, reaching 117 ha in Germany in 2022.^2^

Muscaris was bred at the State Institute of Viticulture Freiburg, Germany, in 1987 from the mother variety Solaris and the father variety Gelber Muskateller, also known as Muscat à petits grains blancs or Muscat Blanc,^3^ with the idea to combine the disease tolerance and environmental adaptability of Solaris with the intense aroma of Gelber Muskateller. Solaris is a white grape variety bred in 1975, whose fungal resistance originates from the wild Asian species *Vitis amurensis*.^3,4^ Actually, it was shown that Muscaris has a similar resistance to downy mildew, powdery mildew, and botrytis as Solaris.^1,5^ Gelber Muskateller is one of the most widely planted white Muscat varieties. It is of Greek origin and has a long cultivation history in Germany. Its fungal resistance is low;^6^ however, it is highly appreciated for its floral and fruity notes, which are intense in the grapes^6,7^ as well as in the wine.^8,9^ Muskateller grapes are often used as a blending partner for other white grape varieties to boost the aroma of wine.^6^

Several studies have been conducted on Muscaris and addressed fungal resistance,^5,10,11^ viticultural characteristics,^12,13^ mechanical grape properties,^13^ and grape composition including phenolic compounds,^14^ minerals, and ascorbic acid.^15^ Further studies focused on the composition of Muscaris must^10,12^ and Muscaris wine.^14,16−20^ However, there is currently no information in the literature on odor-active compounds in Muscaris grapes. Some phenolic compounds including 4-ethenylphenol, 4-ethenyl-2-methoxyphenol, 4-hydroxy-3-methoxybenzaldehyde, 4-hydroxy-3,5-dimethoxybenzaldehyde, and 4-hydroxy-3-ethoxybenzaldehyde were identified as Muscaris grape volatiles, but their odor contribution was not assessed.^14^ We found that the aroma of Muscaris grapes is characterized by a distinct lychee note, the molecular background of which piqued our interest. Lychee notes had previously been reported in Muscaris wine and also in Gewürztraminer wine.^1,21−24^ A comparative gas chromatography–olfactometry (GC–O) analysis applied to Gewürztraminer wine, fresh lychees, and canned lychees detected 12 odorants common to all three samples.^22^ Among them, *cis*-rose oxide, β-damascenone, ethyl 2-methylpropanoate, linalool, and geraniol consistently showed concentrations beyond their odor threshold concentrations (OTCs). *cis*-Rose oxide, linalool, and geraniol had also been reported in odor-active amounts, that is in concentrations exceeding their OTCs, in Muskateller grapes and wines.^8,9,25,26^

Given the gaps in the literature detailed above, our aims were to characterize the major odor-active compounds in Muscaris grapes, compare them with the odor-active compounds in the grapes of its father variety, Muskateller, and elucidate the molecular background of the lychee note in the aroma of the Muscaris grapes. This knowledge is a prerequisite for further research, e.g., on odorant transfer from Muscaris grapes into wine and for targeted breeding. Thus, our study included (i) the screening for odor-active compounds in Muscaris and Muskateller grapes by application of a comparative aroma extract dilution analysis (cAEDA), (ii) the quantitation of potent odorants in grapes of both varieties followed by the calculation of odor activity values (OAVs), (iii) the verification of the analytical data by sensory evaluation of odor reconstitution models, and finally (iv) the identification of the odorants responsible for the characteristic lychee note via omission experiments.

## Materials and Methods

### Grapes

Muscaris and Muskateller grapes were harvested at the State Institute of Viticulture Freiburg, Germany, in early September 2022 and sent refrigerated to the Leibniz-LSB@TUM within 1 day. Unripe and injured berries were removed. The fresh grapes were directly used for odorant screening, as well as for the quantitation of selected compounds. The residual material was shock-frozen with liquid nitrogen and stored in vacuum-sealed bags at −20 °C.

### Reference Odorants

The following reference compounds were purchased at the highest purity available from the following commercial sources: **1**, **2**, **6**–**8**, **11**–**18**, **18a**, **19**, **20**, **22**, **24**, **26**–**29**, **31**, **32**, **34**–**39**, rose oxide (mixture of isomers), citronellal (racemic), (3*S*)-citronellal, and β-citronellol (racemic) (Merck; Darmstadt, Germany), **5** (TCI; Eschborn, Germany), **21** (Thermo Fisher Scientific; Dreieich, Germany), **23** (Chemos; Altdorf, Germany), and **30** (Cayman Chemicals Company; Ann Arbor, MI, USA). Compound **25** was a gift from Symrise (Holzminden, Germany). Compounds **3**, **4**, and **9** were synthesized according to published procedures.^27−29^ Compound **21** was freshly distilled before use. Enantiopure **18b** was obtained from racemic **18** by preparative HPLC.^30^

### Stable Isotopically Substituted Odorants

Compound (^2^H_13–14_)-**5** was purchased from CDN Isotopes (Pointe-Claire, Quebec, Canada). Compounds (^2^H_2_)-**2**, (^2^H_2_)-**4**, (^2^H_2_)-**7**, (^2^H_2–4_)-**8**, (^13^C_5_)-**9**, (^2^H_2–4_)-**10**, (^2^H_3_)-**13**, (^2^H_2_)-**17**, (^2^H_2_)-**18**, (^2^H_2_)-**19**, (^13^C_2_)-**21**, (^2^H_3_)-**23**, (^2^H_2_)-**24**, (^2^H_3–6_)-**25**, (^2^H_2_)-**26**, (^13^C_2_)-**36**, and 2-[(^2^H_3_)methoxy]-4-[(1*E*)-prop-1-en-1-yl]phenol were synthesized as detailed in the references provided in the Table S1.

### Miscellaneous Chemicals

Potassium hydroxide and (2*R*,3*R*)-tartaric acid were purchased from Merck. Dichloromethane (CLN; Freising, Germany) was freshly distilled through a column (120 × 5 cm) packed with Raschig rings prior to use.

### Gas Chromatography

GC–O analyses were performed by using a GC–O/FID instrument. For GC–MS analyses, three different instruments were used: a GC–MS instrument with an ion trap mass spectrometer, a two-dimensional heart-cut GC–GC–HRMS instrument with an orbitrap mass spectrometer, and a comprehensive two-dimensional GC × GC–MS instrument with a time-of-flight (TOF) mass spectrometer. Details of the GC instruments are available in the Supporting Information

### Enantioselective Odorant Analysis

Enantioselective analysis of *cis*-rose oxide, citronellal, linalool, and β-citronellol was accomplished by combining the GC–O/FID instrument and the GC–MS instrument with a chiral GC column. Citronellal and β-citronellol enantiomers were separated with a BGB-174E column, whereas linalool and *cis*-rose oxide enantiomers were separated with a BGB-176 column (both BGB Analytik; Lörrach, Germany). In the case of citronellal, β-citronellol, and linalool enantiomers, the elution order was determined by a comparative analysis of the enantiomeric mixture and at least one of the individual enantiomers. The elution order of *cis*-rose oxide enantiomers was determined from chromatographic and sensory information available in the literature.^31−33^

### Aroma Extract Dilution Analysis

Fresh grapes (200 g) were crushed by hand with the help of a spoon and a kitchen sieve (stainless steel, sieve mesh size of 1 mm), taking care not to break the seeds. After a fixed period (10 min), dichloromethane (400 mL) was added and the mixture was stirred at ambient temperature for 2 h. Skin and seeds were removed with a sieve, and the organic phase was isolated by centrifugation (4500 rpm, 5 °C, 10 min) using a Heraeus Multifuge X3FR (Thermo Fisher Scientific) and dried with anhydrous sodium sulfate. Nonvolatiles were removed by automated solvent-assisted flavor evaporation (aSAFE)^34^ at 40 °C using an open/closed time combination for the pneumatic valve of 0.2 s/10 s. The distillate was concentrated to a final volume of 1.0 mL using a Vigreux column (50 × 1 cm) and a Bemelmans microdistillation device.^35^

The grape volatile isolate was stepwise diluted 1:2 with dichloromethane to obtain dilutions of 1:2, 1:4, 1:8, 1:16, 1:32, 1:64, 1:128, 1:256, 1:512, 1:1024, 1:2048 1:4096, 1:8192, 1:16,384, 1:32,768, and 1:65,536. The undiluted sample, as well as each diluted sample, was subjected to GC–O analysis^36^ using the GC–O/FID instrument detailed in the Supporting Information with the FFAP column. Each odorant was assigned a flavor dilution (FD) factor, representing the dilution factor of the highest diluted sample in which the odorant was detected at the sniffing port during GC–O.

### Odorant Quantitation

The workup of the grapes (20–200 g) basically followed the procedure detailed in the AEDA section. In general, the starting material was frozen-thawed grapes. (3*E*)-Hex-3-enal (**3**) and (3*Z*)-hex-3-enal (**4**) were additionally quantitated in fresh grapes. The stable isotopically substituted odorants used as internal standards (Table S2) were added to the dichloromethane portion used for extraction. Depending on the expected target compound concentrations, amounts of the added internal standards varied between 0.001 and 15 μg. The aSAFE distillates were concentrated to final volumes of 0.1–1.0 mL, and the concentrates were analyzed either with the GC–MS system (**2**–**5**, **7**, **8**, **10**, **13**, **17**–**19**, **21**, **23**, **24**, **26**), the two-dimensional heart-cut GC–GC–HRMS system (**9**, **36**), or the comprehensive two-dimensional GC × GC–MS system (**25**, **34**). All quantitations were carried out in duplicate or triplicate analyses.

Peak areas of the analytes and the respective internal standards were collected from the extracted ion chromatograms using the quantifier ions detailed in Table S2. The concentration of each odorant in the grapes was calculated from the area counts of the analyte peak, the area counts of the internal standard peak, the amount of grapes used in the workup, and the amount of internal standard added by employing a calibration line equation. To obtain the calibration line equation, solutions of the analyte and the respective internal standard were mixed in different concentration ratios and analyzed under the same conditions, followed by linear regression. The calibration line equations are available in Table S2. Individual concentrations and standard deviations can be found in Tables S3–S5.

### Odor Threshold Concentrations

Orthonasal OTCs of (3*E*)-hex-3-enal (**3**), heptanal (**5**), and (2*S*,4*R*)-rose oxide (**10**) were determined in pure water according to the American Society for Testing and Materials (ASTM) standard practice for determination of odor and taste thresholds by a forced-choice ascending concentration series method of limits.^37^ Assessors (6 males and 12–14 females, aged 21–60 years) were recruited from the trained panel of the Leibniz-LSB@TUM. The tests were carried out in separate booths of a room exclusively dedicated to sensory evaluations. The room temperature was 22 ± 2 °C. Further details including the GC–O approach for the purity testing of the odorants prior to their use in the OTC determinations are available in the literature.^38^

### Odor Reconstitution Models

The basis of the odor reconstitution models was aqueous solutions of tartaric acid (3.57 g/L for the Muscaris grape models and 4.23 g/L for the Muskateller grape models). The tartaric acid concentrations in the grapes were previously determined using an enzymatic test kit (R-Biopharm; Darmstadt, Germany). An individual ethanolic stock solution was prepared for each odorant for which an ≥1 OAV had been determined. The absence of odor-active impurities in the reference odorants was checked by GC–O.^38^ Aliquots of the stock solutions were combined and diluted with the appropriate tartaric acid solution to obtain final odorant concentrations in the models that represented the concentrations previously determined in the grapes. Final ethanol concentrations were kept below 200 μL/kg. Using aqueous potassium hydroxide (2 mol/L), the pH of the models was adjusted to 3.8, the value previously determined in both the Muscaris and the Muskateller grapes. The complete odor reconstitution models contained 16 odorants; incomplete models were additionally prepared from which either (2*S*,4*R*)-rose oxide (**10**), geraniol (**26**), or both were omitted.

### Quantitative Olfactory Profiles

Samples (10 g), either freshly crushed frozen-thawed grapes or odor reconstitution models, were placed in cylindrical polytetrafluoroethylene vessels (5.7 cm height, 3.5 cm i.d., 50 mL nominal volume) with lids (Bohlender; Grünsfeld, Germany) and presented to 14 assessors (5 males and 9 females, aged 24–60 years) recruited from the trained panel of the Leibniz-LSB@TUM. The tests were carried out in the room described before. Assessors were asked to orthonasally rate the intensities of nine predefined descriptors on a seven-point scale from 0 to 3 with 0.5 increments and 0 = not detectable, 1 = weak, 2 = moderate, and 3 = strong. Each descriptor was defined by the odor of a reference compound dissolved in water at a concentration 100 times above its respective orthonasal OTC. The nine odor descriptors and the corresponding reference compounds were “green, grassy” (hexanal), “floral” (geraniol), “citrusy” (linalool, racemic), “lychee” ((2*S*,4*R*)-rose oxide), “green apple” ((2*E*)-hex-2-enal), “cucumber” ((2*E*,6*Z*)-nona-2,6-dienal), “mushroom” (oct-1-en-3-one), “honey” (phenylacetaldehyde), and “hay” (3-methylnonane-2,4-dione). Ratings of all assessors were averaged by calculating the arithmetic mean. Statistical analysis was performed with Excel (Microsoft; Redmond, WA, USA).

## Results and Discussion

### Odorant Screening

GC–O in combination with cAEDA applied to the volatile isolates obtained from fresh Muscaris and Muskateller grapes, both of which showed a distinct lychee note, resulted in 39 odor-active compounds, 35 of which were present in both samples. FD factors ranged from 1 to 32,768 for Muscaris grapes and from 1 to 8192 for Muskateller grapes (Table 1). Structure assignments were achieved with the following approach: the RIs obtained for Muscaris and Muskateller grape odorants with two GC columns of different polarity (DB-FFAP and DB-5) in combination with the odor descriptions were compared to published data, foremost those compiled in the Leibniz-LSB@TUM Odorant Database.^39^ The resulting structure proposals were confirmed by GC–O and GC–MS analyses of the grape volatile isolates in parallel with authentic reference compounds. In the case of coelution problems, the comprehensive two-dimensional GC × GC–MS instrument was employed for the mass spectral analyses. For enantiospecific structure assignments, the approach was repeated using two chiral GC columns with differently substituted β-cyclodextrin phases.

**Table 1:** Odorants in the Volatile Isolates Obtained from Muscaris and Muskateller Grapes

no.	odorant^a^	odor^b^	RI^c^		FD factor^d^	
			FFAP	DB-5	Muscaris	Muskateller
**1**	butane-2,3-dione	butter	969	591	8	16
**2**	hexanal	green, grassy	1079	800	16	64
**3**	(3*E*)-hex-3-enal	green, grassy	1134	800	4	4
**4**	(3*Z*)-hex-3-enal	grassy, green	1139	802	128	256
**5**	heptanal	fatty, green	1180	900	32	16
**6**	2-/3-methylbutan-1-ol^g^	malty	1200	735/738	1	2
**7**	(2*E*)-hex-2-enal	green apple	1214	848	4	16
**8**	oct-1-en-3-one	mushroom	1295	981	16	32
**9**	2-acetyl-1-pyrroline^e^	popcorn, roasted	1331	918	32	16
**10**	(2*S*,4*R*)-rose oxide^f^	floral, lychee	1343	1111	64	64
**11**	2,4,5-trimethyl-1,3-thiazole^e^	roasted, earthy	1371	1000	64	64
**12**	acetic acid	vinegar	1447	613	8	2
**13**	3-(methylsulfanyl)propanal^e^	cooked potato	1453	904	512	2048
**14**	(3*R*)-citronellal^f^	citrusy, soapy	1469	1151	16	16
**15**	decanal	citrusy, soapy	1492	1210	8	16
**16**	2-methoxy-3-(2-methylpropyl)pyrazine^e^	bell pepper	1517	1180	8	<1
**17**	(2*E*)-non-2-enal	fatty	1528	1159	32	8
**18**	(3*R*)-/(3*S*)-linalool^f^,^g^	citrusy, floral	1542	1100	64	512
**19**	(2*E*,6*Z*)-nona-2,6-dienal	cucumber	1579	1152	32	32
**20**	undecanal	citrusy, soapy	1598	1308	4	2
**21**	phenylacetaldehyde	floral, honey	1640	1038	128	256
**22**	2-/3-methylbutanoic acid^g^	sweaty	1662	855/866	8	4
**23**	3-methylnonane-2,4-dione^e^	hay, anise, fishy	1700	1243	32	16
**24**	(3*S*)-β-citronellol^f^	soapy, rose	1760	1233	64	8
**25**	(*E*)-β-damascenone^e^	cooked apple	1813	1390	16	32
**26**	geraniol	floral, rose	1844	1262	32,768	8192
**27**	2-methoxyphenol^e^	smoky	1860	1088	8	8
**28**	2-phenylethan-1-ol	honey, floral	1908	1117	4	2
**29**	β-ionone	floral, violet	1932	1488	8	16
**30**	*trans*-4,5-epoxy-(2*E*)-dec-2-enal^e^	metallic	2006	1380	32	16
**31**	4-hydroxy-2,5-dimethylfuran-3(2*H*)-one^e^	caramel	2035	1072	2	1
**32**	octanoic acid	sour, musty	2058	1185	4	4
**33**	unknown	fresh, herb, licorice	2139	1368	4	1
**34**	2-methoxy-4-(prop-2-en-1-yl)phenol	clove	2162	1358	128	<1
**35**	4-ethylphenol	phenolic	2175	1169	4	<1
**36**	sotolon^e^	fenugreek	2198	1110	32	16
**37**	undecanoic acid	soapy, oily	2371	1471	16	4
**38**	phenylacetic acid^e^	floral, honey	2567	1261	1	<1
**39**	4-hydroxy-3-methoxybenzaldehyde	vanilla	2576	1400	4	4

a Odorants showing an FD factor of ≥1 in either of the two samples; odorants were identified by comparing the retention indices on two columns of different polarity (DB-FFAP, DB-5), the mass spectra obtained by GC–MS as well as the odor quality as perceived at the sniffing port during GC–O to data obtained from authentic reference compounds analyzed under equal conditions.

b Odor as perceived at the sniffing port during GC–O.

c Retention index; calculated from the retention time of the odorant and the retention times of adjacent *n*-alkanes by linear interpolation.

d Flavor dilution factor; dilution factor of the highest diluted grape volatile isolate in which the odorant was detected during GC–O by any of three assessors.

e An unequivocal mass spectrum of the compound could not be obtained; identification was based on the remaining criteria detailed in footnote a and by spiking experiments using GC–O/FID.

f Odor-active enantiomers as identified by analysis of the volatile isolates using GC–O/FID in combination with a chiral column.

g The compounds were not separated on the column used for AEDA; the FD factor refers to the mixture.

As a result, the structures of 38 odorants were successfully determined. Only compound **33**, despite all efforts, remained unknown. Among the odorants identified in Muscaris grapes, vanilla-like smelling 4-hydroxy-3-methoxybenzaldehyde (vanillin; **39**) was the only compound which had previously been reported in grapes of this variety.^14^

The compound with by far the highest FD factor among the Muscaris grape odorants was floral, rose-like smelling geraniol (**26**; FD factor 32,768). With 8192, geraniol also showed the highest FD factor among the Muskateller grape odorants. Nineteen odorants were assigned FD factors ≥32 in at least one of the two samples. Twelve of them showed comparable FD factors in both grape varieties. This compound group included green, grassy smelling (3*Z*)-hex-3-enal (**4**; FD factors 128 and 256), floral, honey-like smelling phenylacetaldehyde (**21**; FD factors 128 and 256), floral, lychee-like smelling (2*S*,4*R*)-rose oxide (**10**; FD factors 64 and 64), roasted, earthy smelling 2,4,5-trimethyl-1,3-thiazole (**11**; FD factors 64 and 64), cucumber-like smelling (2*E*,6*Z*)-nona-2,6-dienal (**19**; FD factors 32 and 32), fatty, green smelling heptanal (**5**; FD factors 32 and 16), popcorn-like, roasted smelling 2-acetyl-1-pyrroline (**9**; FD factors 32 and 16), hay-, anise-like, and fishy smelling 3-methylnonane-2,4-dione (**23**; FD factors 32 and 16), metallic smelling *trans*-4,5-epoxy-(2*E*)-dec-2-enal (**30**; FD factors 32 and 16), fenugreek-like smelling sotolon (**36**; FD factors 32 and 16), mushroom-like smelling oct-1-en-3-one (**8**; FD factors 16 and 32), and cooked apple-like smelling (*E*)-β-damascenone (**25**; FD factors 16 and 32). Apart from geraniol, clearly higher FD factors in the Muscaris grapes than in the Muskateller grapes were also obtained for 2-methoxy-4-(prop-2-en-1-yl)phenol (**34**; clove-like; FD factors 128 and <1), (3*S*)-β-citronellol (**24**; soapy, rose-like; FD factors 64 and 8), and (2*E*)-non-2-enal (**17**; fatty; FD factors 32 and 8). In contrast, higher FD factors in the Muskateller grapes were found for cooked potato-like smelling 3-(methylsulfanyl)propanal (**13**; FD factors 512 and 2048), citrusy, floral smelling isomers (3*R*)- and (3*S*)-linalool (**18**; FD factors 64 and 512), and green, grassy smelling hexanal (**2**; FD factors 16 and 64). In summary, however, the odorant spectrum in the Muscaris grapes showed a huge similarity to the odorant spectrum in the Muskateller grapes, thus reflecting the close genetic relationship between the two varieties. This was particularly the case for some monoterpenes considered iconic for Muskateller such as *cis*-rose oxide, linalool, and geraniol. A detailed review on their biosynthesis in grapes has been published by Schwab and Wüst.^40^

### Odorant Quantitation and OAV Calculation

Twenty odorants for which high FD factors in at least one of the two grape samples had been determined during the odorant screening (cf. Table 1) were selected. Quantitation was accomplished by GC–MS using stable isotopically substituted odorants as internal standards. The grape samples subjected to quantitation were from the same batch as those used for odorant screening. Given the time required to collect the screening data and assign the structures, it was impossible to perform the quantitations with fresh material. Accordingly, a majority of odorants were quantitated in frozen-thawed grapes. Exceptions were the lipoxygenase products^41^ (3*Z*)-hex-3-enal (**4**) and (3*E*)-hex-3-enal (**3**), which were quantitated in the fresh grapes in parallel to the screening experiments. This was done because it had been reported that the concentration of (3*Z*)-hex-3-enal can substantially differ between fresh and stored as well as frozen-thawed plant materials.^28,42^

Enantiospecific concentrations of important chiral odorants were calculated from the sum of enantiomers as determined via stable isotopically substituted internal standards in the quantitation assays and the enantiomeric distribution as determined by GC with chiral columns. Chromatograms showing the separation of the enantiomers are available in Figure S1. The enantiomeric distributions of *cis*-rose oxide, linalool, and β-citronellol are listed in Table 2. In both linalool and β-citronellol, the (*S*)-isomer clearly predominated with percentages of 91–98%. With 94 and 99.8%, similar data were reported for linalool in two other Muscat grape varieties.^43^ To the best of our knowledge, the enantiomeric distribution of β-citronellol in Muscat grapes has not been examined so far. In *cis*-rose oxide, the major enantiomer was the odor-active (2*S*,4*R*)-isomer with 71% in the Muscaris grapes and 75% in the Muskateller grapes. In other six Muscat grape varieties previously investigated, the (2*S*,4*R*)-isomer showed even higher percentages of 88–97%.^32^ In total, the data obtained for Muscaris and Muskateller grapes were highly similar, again illustrating their close genetic relation.

**Table 2:** Enantiomeric Distribution of Important Chiral Odorants in Muscaris and Muskateller Grapes

odorant	enantiomer	odor	enantiomeric distribution^a^ (%) in	
			Muscaris	Muskateller
*cis*-rose oxide	2*R*,4*S*	floral, lychee (weak)	29	25
	2*S*,4*R*	floral, lychee (strong)	71	75
linalool	3*R*	citrusy, floral	9	2
	3*S*	citrusy, floral	91	98
β-citronellol	3*R*	soapy, rose	2	3
	3*S*	soapy, rose	98	97

a Mean of triplicates.

The quantitation of the 20 odorants resulted in concentrations between the nanogram per kilogram range and 1160 μg/kg for geraniol (**26**) in Muscaris grapes and 700 μg/kg for (2*E*)-hex-2-enal (**7**) in Muskateller grapes (Table 3). High concentrations of >100 μg/kg were additionally determined for (2*E*)-hex-2-enal (**7**; 325 μg/kg), hexanal (**2**; 294 μg/kg), and (3*S*)-linalool (**18b**; 113 μg/kg) in Muscaris grapes, and hexanal (**2**; 548 μg/kg), (3*S*)-linalool (**18b**; 518 μg/kg), and geraniol (**26**; 442 μg/kg) in Muskateller grapes. Again, the data showed only minor differences between the two varieties.

**Table 3:** Concentrations and OAVs of Important Odorants in Muscaris and Muskateller Grapes

no.^a^	odorant^b^	concentration^c^ (μg/kg)		OTC^d^ (μg/kg)	OAV^e^	
		Muscaris	Muskateller		Muscaris	Muskateller
**26**	geraniol	1160	442	1.1^f^	1100	400
**19**	(2*E*,6*Z*)-nona-2,6-dienal	1.35	0.464	0.0045^f^	300	100
**4**	(3*Z*)-hex-3-enal	21.8	26.6	0.12^f^	180	220
**18a**	(3*R*)-linalool^g^	11.2	10.3	0.087^f^	130	120
**2**	hexanal	294	548	2.4^f^	120	230
**3**	(3*E*)-hex-3-enal	13.7	22.0	0.23^h^	59	95
**18b**	(3*S*)-linalool^g^	113	518	2.7^f^	42	190
**23**	3-methylnonane-2,4-dione	1.23	0.724	0.046^f^	27	16
**8**	oct-1-en-3-one	0.413	0.293	0.016^f^	26	18
**10**	(2*S*,4*R*)-rose oxide^g^	0.873	1.19	0.045^h^	19	26
**24**	(3*S*)-β-citronellol^g^	56.0	28.7	4.9^i^	11	5.9
**17**	(2*E*)-non-2-enal	2.00	1.75	0.19^f^	11	9.2
**21**	phenylacetaldehyde	53.2	38.0	5.2^f^	10	7.3
**13**	3-(methylsulfanyl)propanal	3.66	4.55	0.43^f^	8.5	11
**7**	(2*E*)-hex-2-enal	325	700	110^f^	3.0	6.4
**25**	(*E*)-β-damascenone	0.0106	0.0157	0.0060^f^	1.8	2.6
**34**	2-methoxy-4-(prop-2-en-1-yl)phenol	0.886	0.116	1.8^f^	<1	<1
**5**	heptanal	1.86	1.51	6.1^h^	<1	<1
**36**	sotolon	0.0563	0.0728	1.7^f^	<1	<1
**9**	2-acetyl-1-pyrroline	<0.01	<0.01	0.053^f^	<1	<1

a Numbering according to Table 1.

b Odorants in order of decreasing OAVs in Muscaris grapes.

c Mean of duplicates or triplicates; individual values and standard deviations are available in Tables S3 and S4.

d Orthonasal odor threshold concentration in water.

e Odor activity value; calculated as the ratio of concentration to odor threshold concentration.

f Data taken from the Leibniz-LSB@TUM Odorant Database.^39^

g Concentrations of individual enantiomers were calculated from the concentration of the sum of enantiomers as obtained in the quantitation assays and the enantiomeric distribution depicted in Table 2.

h Data obtained in the current study.^37^

i Data from literature.^44^

To gain more information on the odor activity of the individual odorants, OAVs were calculated as the ratios of the odorant concentrations in the grapes and the OTCs in water. The results (Table 3) revealed an OAV of ≥1 in both grape varieties for 16 of the 20 quantitated odorants. The highest OAV was calculated for geraniol (**26**) in both Muscaris (OAV 1100) and Muskateller (OAV 400) grapes. Whereas in Muscaris grapes no previous data was available in the literature, a huge data set was found for geraniol in Muskateller grapes. The majority of the geraniol concentrations in Muskateller grapes ranged between ∼70^45^ and ∼300 μg/kg,^46^ corresponding to OAVs of ∼60 and ∼300.

Four additional odorants showed an OAV of ≥100 in both grapes in our study, namely, (2*E*,6*Z*)-nona-2,6-dienal (**19**; OAVs 300 and 100), (3*Z*)-hex-3-enal (**4**; OAVs 180 and 220), (3*R*)-linalool (**18a**; OAVs 130 and 120), and hexanal (**2**; OAVs 120 and 230). None of the four compounds had previously been quantitated in Muscaris grapes, but data were particularly available for linalool in Muskateller grapes. Concentrations were typically in the range of ∼300^26^ to ∼600 μg/kg.^47^ However, in no case, the enantiomeric distribution was considered. Given the huge difference in the OTCs of (*R*)- and (*S*)-linalool (cf. Table 3), estimation of the corresponding OAVs was thus not possible.

The OAV range of <100, but >10 in both grapes included five odorants in Table 3. These odorants were (3*E*)-hex-3-enal (**3**; OAVs 59 and 95), (3*S*)-linalool (**18b**; OAVs 42 and 190), 3-methylnonane-2,4-dione (**23**; OAVs 27 and 16), oct-1-en-3-one (**8**; OAVs 26 and 18), and (2*S*,4*R*)-rose oxide (**10**; OAVs 19 and 26). Literature data were available for *cis*-rose oxide in Muskateller grapes. Concentrations varied between ∼0.6^26^ and ∼4 μg/kg^48^ for the enantiomeric mixture. Assuming an enantiomeric distribution of 25/75 as determined by us (cf. Table 2), this would correspond to OAVs of ∼10 to ∼70 for the odor-active (2*S*,4*R*)-rose oxide.

Comparing the OAVs of individual odorants provided in Table 3 between Muscaris and Muskateller grapes resulted in little differences for a majority of compounds; that is, the OAVs did not differ by more than a factor of 2. Important exceptions were geraniol (**26**) and (2*E*,6*Z*)-nona-2,6-dienal (**19**), which not only featured the highest two OAVs in the Muscaris grapes but additionally showed OAVs ∼3 times lower in the Muskateller grapes. Another exception was (3*S*)-linalool (**18b**) with an OAV in the Muskateller grapes almost 5 times higher than that in the Muscaris grapes. However, when the OAVs of both citrusy, floral smelling linalool enantiomers (cf. Table 2) were summed, the combined values differed only by a factor of ∼2 between the Muskateller and the Muscaris grapes.

### Odor Reconstitution and Omission Experiments

**Figure 1:**
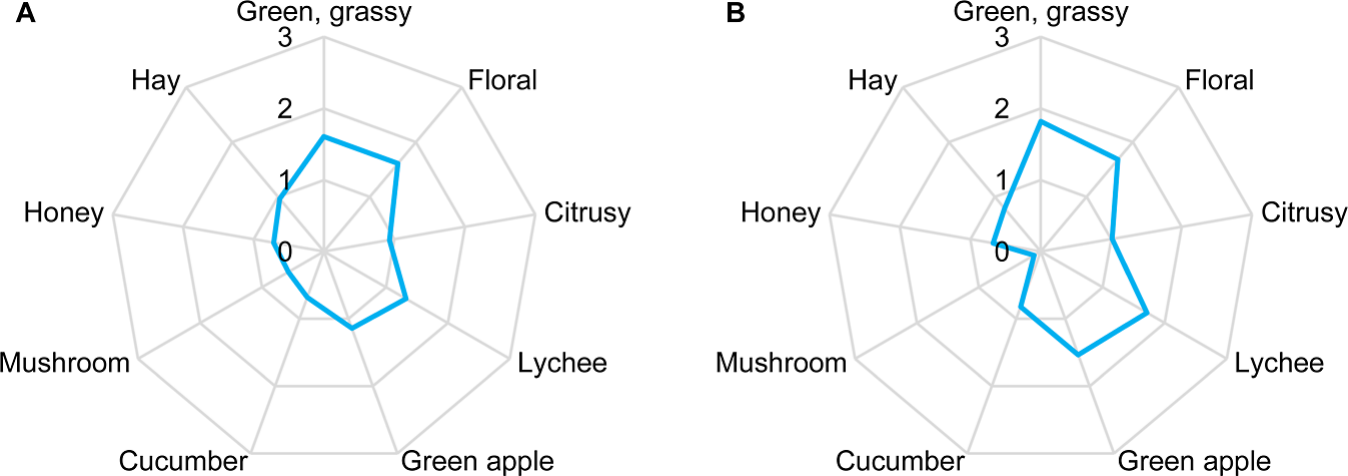
Quantitative olfactory profiles of the odor reconstitution models of Muscaris (A) and Muskateller (B) grapes. Assessors rated the intensity of each descriptor on a scale from 0 to 3 with 0.5 increments and 0 = not detectable, 1 = weak, 2 = moderate, and 3 = strong.

To verify the analytical data and identify the odorants responsible for the characteristic lychee note, odor reconstitution and omission experiments were performed. First, an aqueous odor reconstitution model was prepared for each of the two grape varieties containing all 16 odorants with OAVs ≥1 in the previously determined concentrations (cf. Table 3). Orthonasal evaluation of the two models by a trained sensory panel resulted in quite similar olfactory profiles for both grape varieties. The Muscaris model as well as the Muskateller model showed a balanced combination of green-grassy, floral, lychee, and green apple-like notes (Figure 1). Moreover, the distinct lychee note perceived in the fresh Muscaris grapes was well reflected in the aroma of the Muscaris reconstitution model. A direct comparison of the two reconstitution models with the fresh materials, however, was not possible due to the short shelf life of the grapes. Using fresh grapes of the following harvest as a reference was not considered appropriate as it is known that the vintage can have a huge influence on the grape aroma.^49^

As an alternative to evidence of the correctness of the analytical data, the frozen-thawed Muscaris and Muskateller grapes were used as references for reconstitution models fully based on odorant concentrations in the frozen-thawed materials. This included (3*E*)-hex-3-enal (**3**) and (3*Z*)-hex-3-enal (**4**), which therefore were additionally quantitated in the frozen-thawed grapes (Table S5). The resulting quantitative olfactory profiles (Figure S2) revealed good agreements between the models and the grapes, thus providing evidence that all major aroma-contributing compounds in Muscaris and Muskateller grapes were correctly identified and quantitated.

**Figure 2:**
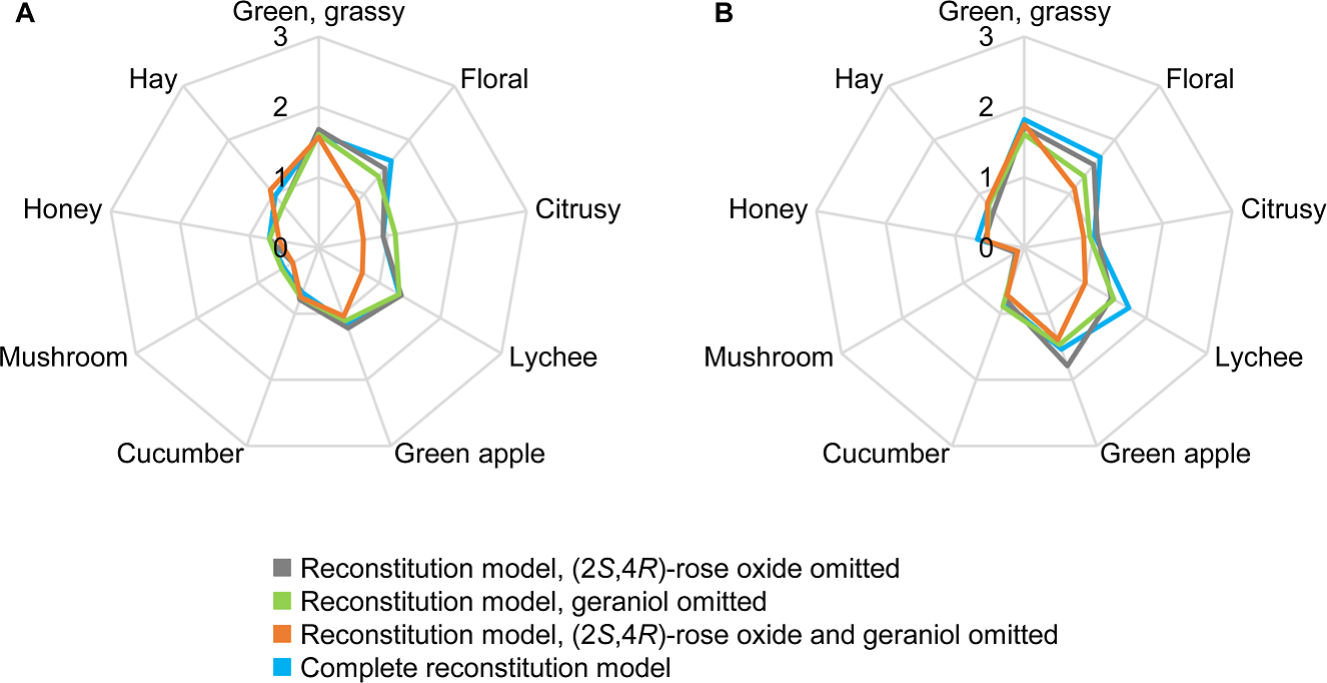
Quantitative olfactory profiles of the odor reconstitution models of Muscaris (A) and Muskateller (B) grapes from which (2*S*,4*R*)-rose oxide, geraniol, or both were omitted. The complete models depicted in Figure 1 are additionally included for comparison. Assessors rated the intensity of each descriptor on a scale from 0 to 3 with 0.5 increments and 0 = not detectable, 1 = weak, 2 = moderate, and 3 = strong.

The primary aim of the omission experiments was to elucidate the molecular basis of the lychee note. The tests were based on the reconstitution models depicted in Figure 1. In the first experiment, (2*S*,4*R*)-rose oxide (**10**) was omitted from the models as it was the only compound among the odorants identified in Muscaris and Muskateller grapes with a specific lychee-like odor (cf. Table 1). The incomplete odor reconstitution models were then compared to the complete odor reconstitution models in quantitative olfactory profile analyses. Surprisingly, the omission of (2*S*,4*R*)-rose oxide did not result in a substantially reduced intensity rating for the lychee note, in either the Muscaris or the Muskateller model (Figure 2). Using paired *t*-tests, *p*-values of 0.8 (Muscaris) and 0.1 (Muskateller) were calculated, suggesting that there was no significant difference in the lychee note. In contrast, when geraniol (**26**) was additionally omitted, the intensity rating for the lychee note dropped considerably: in the Muscaris model by 0.6 units and in the Muskateller model by 0.7 units. The *t*-tests resulted in *p*-values of 0.03 (Muscaris) and 0.01 (Muskateller), suggesting a significant difference between the complete and incomplete reconstitution models. Interestingly, when only geraniol was omitted, the effect on the lychee note was small, similar to the effect observed when only (2*S*,4*R*)-rose oxide was omitted. *p*-Values were 1 in the Muscaris models and 0.2 in the Muskateller models. Thus, it became obvious that the lychee note in the Muscaris grapes, but also in the Muskateller grapes, was generated by the combination of (2*S*,4*R*)-rose oxide and geraniol. Whereas (2*S*,4*R*)-rose oxide showed a specific lychee odor but rather moderate OAVs of 19 and 26 in Muscaris and Muskateller grapes, respectively, geraniol showed high OAVs of 1100 and 400 in combination with a floral and rosy odor not distinctly lychee-like. To the best of our knowledge, the combinatorial effect of (2*S*,4*R*)-rose oxide and geraniol on the lychee note in the aroma of Muscaris and Muskateller grapes has not yet been described in the literature. Further studies are necessary to better understand the interaction of (2*S*,4*R*)-rose oxide and geraniol during olfactory perception.

In summary, this work bridged some of the gaps in the literature. It revealed, for the first time, the major odorants in Muscaris grapes and demonstrated that the combination of (2*S*,4*R*)-rose oxide and geraniol was responsible for the characteristic lychee note in the aroma. These results form the basis for further studies, including research on the transfer of Muscaris grape odorants into wine and their role in the hedonic value of wine. Furthermore, the knowledge of the molecular background of the lychee note may be useful in the targeted breeding of new grape varieties with distinct aroma properties.

## Supporting Information Available

Additional information on GC instruments; references on synthetic procedures to isotopically substituted odorants; stable isotopically substituted internal standards, quantifier ions, and calibration lines used in the quantitation assays; individual odorant concentrations used for mean calculations and standard deviations; chromatograms showing the enantiomeric separation of *cis*-rose oxide, linalool, and β-citronellol by GC–MS with chiral columns; and quantitative olfactory profiles of the frozen-thawed Muscaris and Muskateller grapes in comparison to the quantitative olfactory profiles of the respective odor reconstitution models (PDF)

## Abbreviations

aSAFE: automated solvent-assisted flavor evaporation
ASTM: American Society for Testing and Materials
cAEDA: comparative aroma extract dilution analysis
FD: flavor dilution
FFAP: free fatty acid phase
FID: flame ionization detector
GC: gas chromatography
HR: high resolution
HPLC: high-performance liquid chromatography
i.d.: inner diameter
MS: mass spectrometry
O: olfactometry
OAV: odor activity value
OTC: odor threshold concentration
RI: retention index
TOF: time-of-flight

## Nomenclature

2-acetyl-1-pyrroline: 1-(3,4-dihydro-2*H*-pyrrol-5-yl)ethan-1-one
(3*R*)-/(3*S*)-citronellal: (3*R*)-/(3*S*)-3,7-dimethyloct-6-enal
(3*R*)-/(3*S*)-β-citronellol: (3*R*)-/(3*S*)-3,7-dimethyloct-6-en-1-ol
(*E*)-β-damascenone: (2*E*)-1-(2,6,6-trimethylcyclohexa-1,3-dien-1-yl)but-2-en-1-one
*trans*-4,5-epoxy-(2*E*)-dec-2-enal: (2*E*)-3-[(2*R*,3*R*)/(2*S*,3*S*)-3-pentyloxiran-2-yl]prop-2-enal
geraniol: (2*E*)-3,7-dimethylocta-2,6-dien-1-ol
β-ionone: (3*E*)-4-(2,6,6-trimethylcyclohex-1-en-1-yl)but-3-en-2-one
(3*R*)-/(3*S*)-linalool: (3*R*)-/(3*S*)-3,7-dimethylocta-1,6-dien-3-ol
*cis*-rose oxide: (2*R*,4*S*)-/(2*S*,4*R*)-rose oxide, (2*R*,4*S*)-/(2*S*,4*R*)-4-methyl-2-(2-methylprop-1-en-1-yl)oxane
sotolon: 3-hydroxy-4,5-dimethylfuran-2(5*H*)-one

## References

[ref1] Fungus-resistant grape varieties; State Institute of Viticulture Freiburg, Freiburg, Germany, 2023. https://wbi.landwirtschaft-bw.de/pb/site/pbs-bw-mlr/get/documents_E-1412531467/MLR.LEL/PB5Documents/wbi/051%20Aufgaben%20und%20Fachbereiche/0513%20Fachbereiche/05136%20Referat%20Resistenz%20und%20Klonenz%C3%BCchtung/051325%20Listen%20zu%20PIWI%20Keltertrauben/WBI_Fungus_resistant_grape_varieties_EN.pdf (accessed Nov 7, 2023).

[ref2] Land und Forstwirtschaft, Fischerei: Landwirtschaftliche Bodennutzung-Rebflächen-Fachserie 3 Reihe 3.1.5–2022. Federal Statistical Office (Destatis), Wiesbaden, Germany, 2023. https://www.destatis.de/DE/Themen/Branchen-Unternehmen/Landwirtschaft-Forstwirtschaft-Fischerei/Wein/Publikationen/Downloads-Wein/rebflaechen-2030315227004.pdf?__blob=publicationFile (accessed Nov 7, 2023).

[ref3] Maul E.; Töpfer R.; Röckel F.; Brühl U.; Hundemer M.; Mahler-Ries A.; Walk M.; Kecke S.; Wolck A.; Ganesch A. Vitis International Variety Catalogue. Julius Kühn-Institute (JKI) - Federal Research Center for Cultivated Plants, Institute for Grapevine Breeding Geilweilerhof: Siebeldingen, Germany, 2023. https://www.vivc.de/(accessed Nov 7, 2023).

[ref4] Schwander F.; Eibach R.; Fechter I.; Hausmann L.; Zyprian E.; Töpfer R. Rpv10: a new locus from the Asian Vitis gene pool for pyramiding downy mildew resistance loci in grapevine. Theor. Appl. Genet. 2012, 124, 163–176. 10.1007/s00122-011-1695-4. 21935694

[ref5] Vezzulli S.; Vecchione A.; Stefanini M.; Zulini L. Downy mildew resistance evaluation in 28 grapevine hybrids promising for breeding programs in Trentino region (Italy). Eur. J. Plant Pathol. 2018, 150, 485–495. 10.1007/s10658-017-1298-2.

[ref6] Gelber Muskateller. The Palatinate Magazine, Rohrbach, Germany, 2023. https://www.das-pfalz-magazin.de/weinlexikon/weisse-rebsorten/gelber-muskateller/?L=0 (accessed Nov 7, 2023).

[ref7] Bureau S. M.; Razungles A. J.; Baumes R. L. The aroma of Muscat of Frontignan grapes: effect of the light environment of vine or bunch on volatiles and glycoconjugates. J. Sci. Food Agric. 2000, 80, 2012–2020. 10.1002/1097-0010(200011)80:14<2012::AID-JSFA738>3.0.CO;2-X.

[ref8] Palomo E. S.; Díaz-Maroto M.; Viñas M. G.; Soriano-Pérez A.; Pérez-Coello M. Aroma profile of wines from Albillo and Muscat grape varieties at different stages of ripening. Food Control 2007, 18, 398–403. 10.1016/j.foodcont.2005.11.006.

[ref9] Marcon Â. R.; Delamare A. P. L.; Schwarz L. V.; Pasini L.; Versari A.; Parpinello G. P.; Echeverrigaray S. Volatile and sensory composition of Brazilian Muscat sparkling wine and Asti. J. Food Process. Preserv. 2021, 45, e15240 10.1111/jfpp.15240.

[ref10] Casanova-Gascón J.; Ferrer-Martín C.; Bernad-Eustaquio A.; Elbaile-Mur A.; Ayuso-Rodríguez J. M.; Torres-Sánchez S.; Jarne-Casasús A.; Martín-Ramos P. Behavior of vine varieties resistant to fungal diseases in the Somontano region. Agronomy 2019, 9, 738 10.3390/agronomy9110738.

[ref11] Zini E.; Dolzani C.; Stefanini M.; Gratl V.; Bettinelli P.; Nicolini D.; Betta G.; Dorigatti C.; Velasco R.; Letschka T.; Vezzulli S. R-loci arrangement versus downy and powdery mildew resistance level: a Vitis hybrid survey. Int. J. Mol. Sci. 2019, 20, 3526 10.3390/ijms20143526. 31323823 PMC6679420

[ref12] Pedò S.; Bottura M.; Porro D. Development, yield potential and nutritional aspects of resistant grapevine varieties in Trentino Alto Adige. BIO Web of Conferences; Poni S., Ed.; EDP Sciences S.A.: Les Ulis, France, 2019; Vol. 13, p 02004.

[ref13] Porro D.; Wolf M.; Pedò S. Evaluation of mechanical properties of berries on resistant or tolerant varieties of grapevine. BIO Web of Conferences; Poni S., Ed.; EDP Sciences S.A.: Les Ulis, France, 2019; Vol. 13, p 01005.

[ref14] Barnaba C.; Dellacassa E.; Nicolini G.; Giacomelli M.; Roman Villegas T.; Nardin T.; Larcher R. Targeted and untargeted high resolution mass approach for a putative profiling of glycosylated simple phenols in hybrid grapes. Food Res. Int. 2017, 98, 20–33. 10.1016/j.foodres.2017.01.011. 28610729

[ref15] Czaplicka M.; Parypa K.; Szewczuk A.; Gudarowska E.; Rowinska M.; Zubaidi M. A.; Nawirska-Olszanska A. Assessment of selected parameters for determining the internal quality of white grape cultivars grown in cold climates. Appl. Sci. 2022, 12, 5534 10.3390/app12115534.

[ref16] Leis D.; Renner W.; Leitner E. Characterisation of wines produced from fungus resistant grape varieties. In Flavour Science, Proceedings of the XV Weurman Flavour Research Symposium; Siegmund B., Leitner E., Eds.; Verlag der Technischen Universität Graz: Graz, Austria, 2018; pp 511–514.

[ref17] Román T.; Nicolini G.; Barp L.; Malacarne M.; Tait F.; Larcher R. Shikimic acid concentration in white wines produced with different processing protocols from fungus-resistant grapes growing in the Alps. Vitis 2018, 57, 41–46. 10.5073/vitis.2018.57.41-46.

[ref18] Ruocco S.; Perenzoni D.; Angeli A.; Stefanini M.; Rühl E.; Patz C. D.; Mattivi F.; Rauhut D.; Vrhovsek U. Metabolite profiling of wines made from disease-tolerant varieties. Eur. Food Res. Technol. 2019, 245, 2039–2052. 10.1007/s00217-019-03314-z.

[ref19] van Wyk N.; Pretorius I. S.; von Wallbrunn C. Assessing the oenological potential of *Nakazawaea ishiwadae*, *Candida railenensis* and *Debaryomyces hansenii* strains in mixed-culture grape must fermentation with *Saccharomyces cerevisiae*. Fermentation 2020, 6, 49 10.3390/fermentation6020049.

[ref20] van Wyk N.; Scansani S.; Beisert B.; Brezina S.; Fritsch S.; Semmler H.; Pretorius I. S.; Rauhut D.; von Wallbrunn C. The use of *Hanseniaspora occidentalis* in a sequential must inoculation to reduce the malic acid content of wine. Appl. Sci. 2022, 12, 6919 10.3390/app12146919.

[ref21] Reynolds A. G.; Wardle D. A.; Dever M. Vine performance, fruit composition, and wine sensory attributes of Gewürztraminer in response to vineyard location and canopy manipulation. Am. J. Enol. Vitic. 1996, 47, 77–92. 10.5344/ajev.1996.47.1.77.

[ref22] Ong P. K. C.; Acree T. E. Similarities in the aroma chemistry of Gewürztraminer variety wines and lychee (*Litchi chinesis* Sonn.) fruit. J. Agric. Food Chem. 1999, 47, 665–670. 10.1021/jf980452j. 10563950

[ref23] Rusjan D.; Strlič M. S.; Košmerl T.; Prosen H. The response of monoterpenes to different enzyme preparations in Gewürztraminer (*Vitis vinifera* L.) wines. S. Afr. J. Enol. Vitic. 2009, 30, 56–64. 10.21548/30-1-1425.

[ref24] Katarína F.; Katarína M.; Katarína Ď.; Ivan Š.; Fedor M. Influence of yeast strain on aromatic profile of Gewürztraminer wine. LWT—Food Sci. Technol. 2014, 59, 256–262. 10.1016/j.lwt.2014.05.057.

[ref25] Čuš F.; Zabukovec P.; Schroers H. Indigenous yeasts perform alcoholic fermentation and produce aroma compounds in wine. Czech J. Food Sci. 2017, 35, 329–345. 10.17221/398/2016-CJFS.

[ref26] Li X. Y.; Wen Y. Q.; Meng N.; Qian X.; Pan Q. H. Monoterpenyl glycosyltransferases differentially contribute to production of monoterpenyl glycosides in two aromatic *Vitis vinifera* varieties. Front. Plant Sci. 2017, 8, 1226 10.3389/fpls.2017.01226. 28751905 PMC5508019

[ref27] Steinhaus M.; Sinuco D.; Polster J.; Osorio C.; Schieberle P. Characterization of the aroma-active compounds in pink guava (*Psidium guajava*, L.) by application of the aroma extract dilution analysis. J. Agric. Food Chem. 2008, 56, 4120–4127. 10.1021/jf8005245. 18476695

[ref28] Kreissl J.; Schieberle P. Characterization of aroma-active compounds in Italian tomatoes with emphasis on new odorants. J. Agric. Food Chem. 2017, 65, 5198–5208. 10.1021/acs.jafc.7b01108. 28573856

[ref29] Buttery R. G.; Ling L. C.; Juliano B. O.; Turnbaugh J. G. Cooked rice aroma and 2-acetyl-1-pyrroline. J. Agric. Food Chem. 1983, 31, 823–826. 10.1021/jf00118a036.

[ref30] Reglitz K.; Stein J.; Ackermann J.; Heigl V.; Brass L.; Ampenberger F.; Zarnkow M.; Steinhaus M. Enantiospecific determination of the odour threshold concentrations of (*R*)- and (*S*)-linalool in water and beer. Brew. Sci. 2023, 76, 92–96.

[ref31] Wüst M.; Beck T.; Mosandl A. Conversion of citronellyl diphosphate and citronellyl β-D-glucoside into rose oxide by *Pelargonium graveolens*. J. Agric. Food Chem. 1999, 47, 1668–1672. 10.1021/jf980972e. 10564036

[ref32] Luan F.; Mosandl A.; Gubesch M.; Wüst M. Enantioselective analysis of monoterpenes in different grape varieties during berry ripening using stir bar sorptive extraction- and solid phase extraction-enantioselective-multidimensional gas chromatography-mass spectrometry. J. Chromatogr. A 2006, 1112, 369–374. 10.1016/j.chroma.2005.12.056. 16405900

[ref33] Wüst M.; Mosandl A. Important chiral monoterpenoid ethers in flavours and essential oils -enantioselective analysis and biogenesis. Eur. Food Res. Technol. 1999, 209, 3–11. 10.1007/s002170050447.

[ref34] Schlumpberger P.; Stübner C. A.; Steinhaus M. Development and evaluation of an automated solvent-assisted flavour evaporation (aSAFE). Eur. Food Res. Technol. 2022, 248, 2591–2602. 10.1007/s00217-022-04072-1.

[ref35] Bemelmans J. M. H. Review of isolation and concentration techniques. In Progress in Flavour Research; Land G. G., Nursten H. E., Eds.; Applied Science Publishers: London, UK, 1979, pp 79–88.

[ref36] Steinhaus M. Gas chromatography-olfactometry: principles, practical aspects and applications in food analysis. In Advanced Gas Chromatography in Food Analysis; Tranchida P., Ed.; The Royal Society of Chemistry: Cambridge, UK, 2019; pp 337–399.

[ref37] ASTM International. E679–19 Standard Practice for Determination of Odor and Taste Thresholds by a Forced-Choice Ascending Concentration Series Method of Limits; ASTM: West Conshohocken, PA, 2019.

[ref38] Czerny M.; Christlbauer M.; Christlbauer M.; Fischer A.; Granvogl M.; Hammer M.; Hartl C.; Hernandez N.; Schieberle P. Re-investigation on odour thresholds of key food aroma compounds and development of an aroma language based on odour qualities of defined aqueous odorant solutions. Eur. Food Res. Technol. 2008, 228, 265–273. 10.1007/s00217-008-0931-x.

[ref39] Kreissl J.; Mall V.; Steinhaus P.; Steinhaus M. Leibniz-LSB@TUM Odorant Database, Version 1.2; Leibniz Institute for Food Systems Biology at the Technical University of Munich: Freising, Germany, 2022. https://www.leibniz-lsb.de/en/databases/leibniz-lsbtum-odorant-database (accessed Nov 7, 2023).

[ref40] Schwab W.; Wüst M. Understanding the constitutive and induced biosynthesis of mono-and sesquiterpenes in grapes (*Vitis vinifera*): a key to unlocking the biochemical secrets of unique grape aroma profiles. J. Agric. Food Chem. 2015, 63, 10591–10603. 10.1021/acs.jafc.5b04398. 26592256

[ref41] Kalua C. M.; Boss P. K. Evolution of volatile compounds during the development of Cabernet Sauvignon grapes (*Vitis vinifera* L.). J. Agric. Food Chem. 2009, 57, 3818–3830. 10.1021/jf803471n. 19309150

[ref42] Sellami I.; Mall V.; Schieberle P. Changes in the key odorants and aroma profiles of hamlin and valencia orange juices not from concentrate (NFC) during chilled storage. J. Agric. Food Chem. 2018, 66, 7428–7440. 10.1021/acs.jafc.8b02257. 29889522

[ref43] Luan F.; Hampel D.; Mosandl A.; Wüst M. Enantioselective analysis of free and glycosidically bound monoterpene polyols in *Vitis vinifera* L. cvs. Morio Muscat and Muscat Ottonel: evidence for an oxidative monoterpene metabolism in grapes. J. Agric. Food Chem. 2004, 52, 2036–2041. 10.1021/jf030701q. 15053548

[ref44] Schaller T.; Schieberle P. Quantitation of key aroma compounds in fresh, raw ginger (*Zingiber officinale* Roscoe) from China and roasted ginger by stable isotope dilution assays and aroma profiling by recombination experiments. J. Agric. Food Chem. 2020, 68, 15284–15291. 10.1021/acs.jafc.0c06733. 33300793

[ref45] Río Segade S.; Vilanova M.; Pollon M.; Giacosa S.; Torchio F.; Rolle L. Grape VOCs response to postharvest short-term ozone treatments. Front. Plant Sci. 2018, 9, 1826 10.3389/fpls.2018.01826. 30619399 PMC6297214

[ref46] Ribéreau-Gayon P.; Boidron J. N.; Terrier A. Aroma of Muscat grape varieties. J. Agric. Food Chem. 1975, 23, 1042–1047. 10.1021/jf60202a050.

[ref47] Gunata Y. Z.; Bayonove C. L.; Baumes R. L.; Cordonnier R. E. The aroma of grapes. Localisation and evolution of free and bound fractions of some grape aroma components cv Muscat during first development and maturation. J. Sci. Food Agric. 1985, 36, 857–862. 10.1002/jsfa.2740360915.

[ref48] Matarese F.; Cuzzola A.; Scalabrelli G.; D’Onofrio C. Expression of terpene synthase genes associated with the formation of volatiles in different organs of *Vitis vinifera*. Phytochemistry 2014, 105, 12–24. 10.1016/j.phytochem.2014.06.007. 25014656

[ref49] Robinson A. L.; Boss P. K.; Solomon P. S.; Trengove R. D.; Heymann H.; Ebeler S. E. Origins of grape and wine aroma. Part 1. Chemical components and viticultural impacts. Am. J. Enol. Vitic. 2014, 65, 1–24. 10.5344/ajev.2013.12070.

