# Supplementary material for: Molecular Background of the Lychee Aroma of Vitis vinifera L. ‘Muscaris’
Source: J Agric Food Chem. 2024 Jan 12;72(3):1674–82. doi: 10.1021/acs.jafc.3c08298 (PMC10811687; doi:10.1021/acs.jafc.3c08298)
Supplement: Supplementary file 1 — jf3c08298_si_001.pdf [file jf3c08298_si_001.pdf]

Supporting Information

Molecular Background of the Lychee Aroma of *Vitis vinifera* L. 'Muscaris'

Xingjie Wang,[1,2] Stephanie Frank,[2,*] and Martin Steinhaus[1,2,*]

[1] Technical University of Munich, TUM School of Natural Sciences, Department of Chemistry, Lichtenbergstraße 4, 85748 Garching, Germany

[2] Leibniz Institute for Food Systems Biology at the Technical University of Munich (Leibniz-LSB@TUM), Lise-Meitner-Straße 34, 85354 Freising, Germany

*E-mail: s.frank.leibniz-lsb@tum.de, martin.steinhaus@tum.de

Overview

Additional Information on GC Instruments

GC–O/FID Instrument

GC–MS Instrument

Heart-Cut GC–GC–HRMS Instrument

Comprehensive Two-Dimensional GC×GC–MS Instrument

Additional Tables

Table S1. References on Synthetic Procedures to Isotopically Substituted Odorants

Table S2. Stable Isotopically Substituted Internal Standards, Quantifier Ions, and Calibration Lines Used in the Quantitation Assays

Table S3. Concentrations of Important Odorants in Muscaris Grapes

Table S4. Concentrations of Important Odorants in Muskateller Grapes

Table S5. Concentrations of (3*E*)-Hex-3-enal and (3*Z*)-Hex-3-enal in the Frozen-Thawed Muscaris and Muskateller Grapes

Additional Figure

Figure S1. Enantiomeric separation of *cis*-rose oxide (A), linalool (B), and *β*-citronellol (C) by GC–MS with chiral columns.

Figure S2. Quantitative olfactory profiles of the frozen-thawed Muscaris (A) and Muskateller (B) grapes in comparison to the quantitative olfactory profiles of the respective odor reconstitution models.

GC–O/FID Instrument

A Trace Gas Chromatograph Ultra (Thermo Fisher Scientific; Dreieich, Germany) was equipped with a cold on-column injector, a flame ionization detector (FID), and a sniffing-port custom-made from aluminum as detailed in *J. Agric. Food Chem*. **2008**, *56*, 4120–4127. The column was either a DB-FFAP column, 30 m × 0.32 mm i.d., 0.25 µm film thickness (Agilent; Waldbronn, Germany), a DB-5 column, 30 m × 0.32 mm i.d., 0.25 µm film thickness (Agilent), a BGB-174E column, 30 m × 0.25 mm i.d., 0.25 µm film thickness (BGB Analytik; Lörrach, Germany), or a BGB-176 column, 30 m × 0.25 mm i.d., 0.25 µm film thickness (BGB Analytik). The carrier gas was helium at 60 kPa (DB-FFAP), 46 kPa (DB-5), 142 kPa (BGB-174E), and 139 kPa (BGB-176) constant pressure. The injection volume was 1 µL. For DB-FFAP and DB-5, the initial oven temperature was 40 °C for 2 min. Then the temperature was ramped at 6 °C/min to 230 °C (DB-FFAP) or to 240 °C (DB-5), which was held for 5 min. For BGB-174E, the initial oven temperature was 40 °C for 2 min. Then the temperature was ramped at 10 °C/min to 90 °C, held for 5 min, then ramped at 0.5 °C /min to 105 °C, and finally ramped at 10 °C /min to 200 °C, which was held for 5 min. For BGB-176, the initial oven temperature was 40 °C for 2 min. Then the temperature was ramped at 2 °C/min to 200 °C, which was held for 5 min. A Y-shaped glass splitter connected the end of the column with two uncoated but deactivated fused silica capillaries, each 50 cm × 0.25 mm i.d., which delivered the column effluent in two equal parts to the FID (250 °C base temperature) and the sniffing port (230 °C base temperature), respectively. For GC–O analysis, a trained assessor placed the nose directly above the sniffing port and evaluated the effluent. Whenever an odor was detected, the position, as well as the odor quality, were marked in the FID chromatogram. For each odorant, a retention index was calculated by linear interpolation from its retention time and the retention times of adjacent *n*-alkanes as detailed in *J. Chromatogr. A*. **1963**, *11*, 463–471.

GC–MS Instrument

A 7890B gas chromatograph (Agilent) was equipped with a GC 80 autosampler and a multimode injector. The column was either a DB-FFAP column, 30 m × 0.25 mm i.d., 0.25 µm film thickness (Agilent), a BGB-174E column, 30 m × 0.25 mm i.d., 0.25 µm film thickness (BGB Analytik), or a BGB-176 column, 30 m × 0.25 mm i.d., 0.25 µm film thickness (BGB Analytik). The carrier gas was helium at 1.0 mL/min constant flow. The injection volume was 1 µL or 2 µL. For DB-FFAP, the initial oven temperature was 40 °C for 2 min. Then the temperature was ramped at 6 °C/min [(3*E*)-hex-3-enal and (3*Z*)-hex-3-enal] or 8 °C/min (all other odorants) to 230 °C, which was held for 5 min. For BGB-174E and BGB-176, the oven temperature programs were identical to those detailed in the GC–O/FID instrument section. The GC was connected to a Saturn 240 ion trap mass spectrometer (Agilent) operated in the chemical ionization (CI) mode with methanol as reagent gas and a scan range of *m/z* 60–250. Data were analyzed with the MS Workstation software (Agilent).

Heart-Cut GC–GC–HRMS Instrument

A Trace 1310 gas chromatograph (Thermo Fisher Scientific) was equipped with a TriPlus RSH autosampler, a programmed temperature vaporizing (PTV) injector, an FID (250 °C base temperature), and a custom-made sniffing port (cf. *J. Agric. Food Chem*. **2008**, *56*, 4120–4127; 230 °C base temperature). The column was a DB-FFAP column, 30 m × 0.25 mm i.d., 0.25 µm film thickness (Agilent). The carrier gas was helium at 1.0 mL/min constant flow. The injection volume was 1 µL or 2 µL. The initial oven temperature was 40 °C for 2 min. Then the temperature was ramped at 6 °C/min to 230 °C, which was held for 5 min. The end of the column was connected to a Deans switch (Trajan; Ringwood, Australia) used for heart-cutting. The Deans switch directed the eluate of the column via uncoated but deactivated fused silica capillaries (0.1 mm i.d.) time-programmed either simultaneously to the FID and the sniffing port used as monitor detectors or to a second GC column, which was a DB-1701 column, 30 m × 0.25 mm i.d., 0.25 µm film thickness (Agilent). This column was installed in a second Trace 1310 gas chromatograph. The capillary

to the second column first passed through a heated (250 °C) hose connecting the two gas chromatographs and then through a liquid nitrogen-cooled trap used to refocus the heart-cut. The initial temperature of the second oven was 40 °C for 2 min. Then the temperature was ramped at 6 °C/min to 240 °C, which was held for 5 min. The end of the second column was connected to a Q Exactive GC orbitrap mass spectrometer (Thermo Fisher Scientific) operated in the high-resolution CI mode with isobutane as reagent gas and a scan range of *m/z* 90–150. Data were analyzed with the Xcalibur software (Thermo Fisher Scientific).

Comprehensive Two-Dimensional GC×GC–MS Instrument

A 6890 Plus gas chromatograph (Agilent) was equipped with a GC PAL autosampler (CTC Analytics; Zwingen, Switzerland) and a CIS 4 injector (Gerstel; Mülheim an der Ruhr, Germany). The column in the first dimension was a DB-FFAP column, 30 m × 0.25 mm i.d., 0.25 µm film thickness (Agilent). The carrier gas was helium at 2.0 mL/min constant flow. The injection volume was 2 µL. The initial oven temperature was 40 °C for 2 min. Then the temperature was ramped at 6 °C/min to 230 °C, which was held for 5 min. The end of the first column was connected via a liquid nitrogen-cooled dual-stage quad-jet modulator (Leco; Mönchengladbach, Germany) to a DB-5 column, 3 m × 0.15 mm i.d., 0.30 µm film thickness (Agilent) inside the secondary oven, which was mounted inside the primary GC oven. The modulation time was 4 s. The initial temperature of the second oven was 70 °C for 2 min. Then the temperature was ramped at 6 °C/min to 250 °C, which was held for 5 min. The end of the second column was connected to a Pegasus III TOF mass spectrometer (Leco) operated in the electron ionization (EI) mode at 70 eV with a scan range of *m/z* 35–350 and a scan rate of 100 spectra/s. Data were analyzed with the GC Image software (GC Image; Lincoln, Nebraska, USA).

Table S1. References on Synthetic Procedures to Isotopically Substituted Odorants

compound	reference
($^{2}H_{4}$)-**2**	*J. Agric. Food Chem.* **2009**, *57*, 2882–2888.
($^{2}H_{2}$)-**4**	*J. Agric. Food Chem.* **2022**, *70*, 13979–13986.
($^{2}H_{2}$)-**7**	*J. Agric. Food Chem.* **2019**, *67*, 5838–5846; using hex-2-yn-1-ol as educt.
($^{2}H_{2–4}$)-**8**	*Lebensm.-Wiss. Technol.* **1990**, *23*, 513–522.
($^{13}C_{5}$)-**9**	*J. Agric. Food Chem.* **2013**, *61*, 5226–5235.
($^{2}H_{2–4}$)-**10**	*J. Agric. Food Chem.* **1997**, *45*, 3027–3032.
($^{2}H_{3}$)-**13**	*J. Agric. Food Chem.* **2019**, *67*, 5838–5846.
($^{2}H_{2}$)-**17**	*J. Agric. Food Chem.* **2019**, *67*, 5838–5846.
($^{2}H_{2}$)-**18**	*J. Agric. Food Chem.* **2003**, *51*, 7100–7105.
($^{2}H_{2}$)-**19**	*Lebensm.-Wiss. Technol.* **1990**, *23*, 513–522.
($^{13}C_{2}$)-**21**	*J. Agric. Food Chem.* **2006**, *54*, 916–924.
($^{2}H_{3}$)-**23**	*Lebensm.-Wiss. Technol.* **1990**, *23*, 513–522.
($^{2}H_{2}$)-**24**	*J. Agric. Food Chem.* **2020**, *68*, 15284–15291.
($^{2}H_{3–6}$)-**25**	*J. Agric. Food Chem.* **1991**, *39*, 757–759.
($^{2}H_{2}$)-**26**	*Eur. Food Res. Technol*. **2009**, *229*, 319–328.
($^{13}C_{2}$)-**36**	Blank, I. et al. In *Progress in Flavour Precursor Studies*; Schreier, P., Winterhalter, P., Eds.; Allured Publishing: Carol Stream, IL, 1993; pp. 103–109.
($^{2}H_{3}$)-MPP[a]	*J. Agric. Food Chem.* **2020**, *68*, 15284–15291.

[a]($^{2}H_{3}$)-2-methoxy-4-[(1*E*)-prop-1-en-1-yl]phenol.

Table S2. Stable Isotopically Substituted Internal Standards, Quantifier Ions, and Calibration Lines Used in the Quantitation Assays

odorant	internal standard	quantifier ions (*m*/*z*)		calibration line equation[a]	R^2
		analyte	standard		
**2**	($^2H_4$)-**2**	101	105	y = 1.1549x + 0.0436	1.000
**3**	($^2H_2$)-**4**	81	83	y = 0.8761x − 0.1416	0.996
**4**	($^2H_2$)-**4**	81	83	y = 0.9199x + 0.0248	1.000
**5**	($^2H_{13–14}$)-**5**	115	128–129	y = 0.8434x − 0.1115	0.999
**7**	($^2H_2$)-**7**	99	101	y = 0.8290x + 0.0526	1.000
**8**	($^2H_{2–4}$)-**8**	127	129–131	y = 0.9572x − 0.0355	1.000
**9**	($^{13}C_5$)-**9**	112	117	y = 1.1438x − 0.1186	0.995
**10**	($^2H_{2–4}$)-**10**	137	139–141	y = 0.8375x + 0.0478	1.000
**13**	($^2H_3$)-**13**	105	108	y = 1.0144x − 0.0102	1.000
**17**	($^2H_2$)-**17**	141	143	y = 0.8422x − 0.0195	0.999
**18**	($^2H_2$)-**18**	137	139	y = 0.8402x + 0.0362	1.000
**19**	($^2H_2$)-**19**	139	141	y = 0.7638x + 0.0784	0.999
**21**	($^{13}C_2$)-**21**	121	123	y = 0.9953x + 0.0284	1.000
**23**	($^2H_3$)-**23**	171	174	y = 0.9392x + 0.0160	1.000
**24**	($^2H_2$)-**24**	157	159	y = 0.9616x + 0.0308	0.999
**25**	($^2H_{3–6}$)-**25**	121	124–127	y = 0.8440x + 0.0126	1.000
**26**	($^2H_2$)-**26**	137	139	y = 1.1121x − 0.0765	1.000
**34**	($^2H_3$)-MPP[b]	164	167	y = 0.8451x − 0.0405	0.999
**36**	($^{13}C_2$)-**36**	129	131	y = 1.0904x − 0.0002	1.000

[a]y = peak area counts standard / peak area counts analyte; x = concentration standard (µg/mL) / concentration analyte (µg/mL). [b]($^2H_3$)-2-methoxy-4-[(1*E*)-prop-1-en-1-yl]phenol.

Table S3. Concentrations of Important Odorants in Muscaris Grapes

odorant	concentration (µg/kg)			
	experiment 1	experiment 2	experiment 3	mean ± SD (CV)[a]
**2**	249	329	305	294 ± 41 (14%)
**3**	15.5	13.2	12.3	13.7 ± 1.6 (12%)
**4**	22.7	23.1	19.5	21.8 ± 2.0 (9%)
**5**	1.80	1.87	1.91	1.86 ± 0.06 (3%)
**7**	350	294	331	325 ± 29 (9%)
**8**	0.329	0.454	0.456	0.413 ± 0.073 (18%)
**9**	≤0.00693[b]	≤0.00617[b]	≤0.00590[b]	
**10**	0.826	0.827	0.967	0.873 ± 0.081 (9%)
**13**	3.38	3.77	3.82	3.66 ± 0.24 (7%)
**17**	2.04	2.04	1.93	2.00 ± 0.07 (3%)
**18a**	12.2	9.89	11.5	11.2 ± 1.2 (10%)
**18b**	123	100	116	113 ± 12 (10%)
**19**	1.27	1.45	1.31	1.35 ± 0.09 (7%)
**21**	48.8	60.5	50.4	53.2 ± 6.3 (12%)
**23**	1.39	1.25	1.06	1.23 ± 0.16 (13%)
**24**	56.4	57.2	54.3	56.0 ± 1.5 (3%)
**25**	0.00982	0.0114		0.0106 ± 0.0011 (10%)
**26**	1220	1120	1150	1160 ± 50 (5%)
**34**	0.939	0.934	0.786	0.886 ± 0.087 (10%)
**36**	0.0550	0.0616	0.0522	0.0563 ± 0.0048 (9%)

[a]SD, standard deviation; CV, coefficient of variation. [b]No analyte peak was observed; values were derived from the integration of the background noise.

Table S4. Concentrations of Important Odorants in Muskateller Grapes

odorant	concentration (µg/kg)			
	experiment 1	experiment 2	experiment 3	mean ± SD (CV)[a]
**2**	519	488	638	548 ± 79 (14%)
**3**	23.7	20.2		22.0 ± 2.5 (11%)
**4**	31.3	26.1	22.5	26.6 ± 4.4 (17%)
**5**	1.54	1.60	1.40	1.51 ± 0.10 (7%)
**7**	823	676	601	700 ± 113 (16%)
**8**	0.322	0.291	0.267	0.293 ± 0.028 (9%)
**9**	≤0.00737[b]	≤0.00609[b]		
**10**	1.17	1.19	1.21	1.19 ± 0.02 (1%)
**13**	4.14	4.53	5.00	4.55 ± 0.43 (10%)
**17**	1.87	1.65	1.72	1.75 ± 0.11 (6%)
**18a**	11.6	10.4	8.93	10.3 ± 1.3 (13%)
**18b**	582	523	449	518 ± 66 (13%)
**19**	0.425	0.435	0.532	0.464 ± 0.059 (13%)
**21**	34.7	36.5	42.9	38.0 ± 4.3 (11%)
**23**	0.787	0.712	0.675	0.724 ± 0.057 (8%)
**24**	28.0	30.7	27.3	28.7 ± 1.8 (6%)
**25**	0.0145	0.0169		0.0157 ± 0.0016 (10%)
**26**	469	412	446	442 ± 29 (6%)
**34**	0.119	0.125	0.103	0.116 ± 0.011 (10%)
**36**	0.0694	0.0702	0.0789	0.0728 ± 0.0053 (7%)

[a]SD, standard deviation; CV, coefficient of variation. [b]No analyte peak was observed; values were derived from the integration of the background noise.

Table S5. Concentrations of (3*E*)-Hex-3-enal and (3*Z*)-Hex-3-enal in the Frozen-Thawed Muscaris and Muskateller Grapes

variety	odorant	concentration (µg/kg)			
		experiment 1	experiment 2	experiment 3	mean ± SD (CV)[a]
Muscaris	**3**	5.96	6.09		6.02 ± 0.09 (1%)
	**4**	151	150	134	145 ± 9 (6%)
Muskateller	**3**	10.9	14.2	18.1	14.4 ± 2.3 (16%)
	**4**	81.0	79.8	73.1	78.0 ± 4.2 (5%)

[a]SD, standard deviation; CV, coefficient of variation.

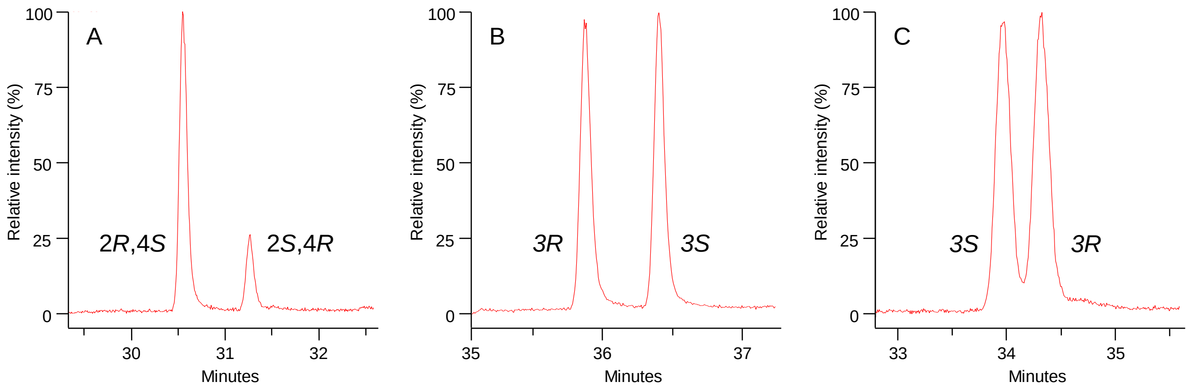

Figure S1. Enantiomeric separation of *cis*-rose oxide (A), linalool (B), and *β*-citronellol (C) by GC–MS with chiral columns.

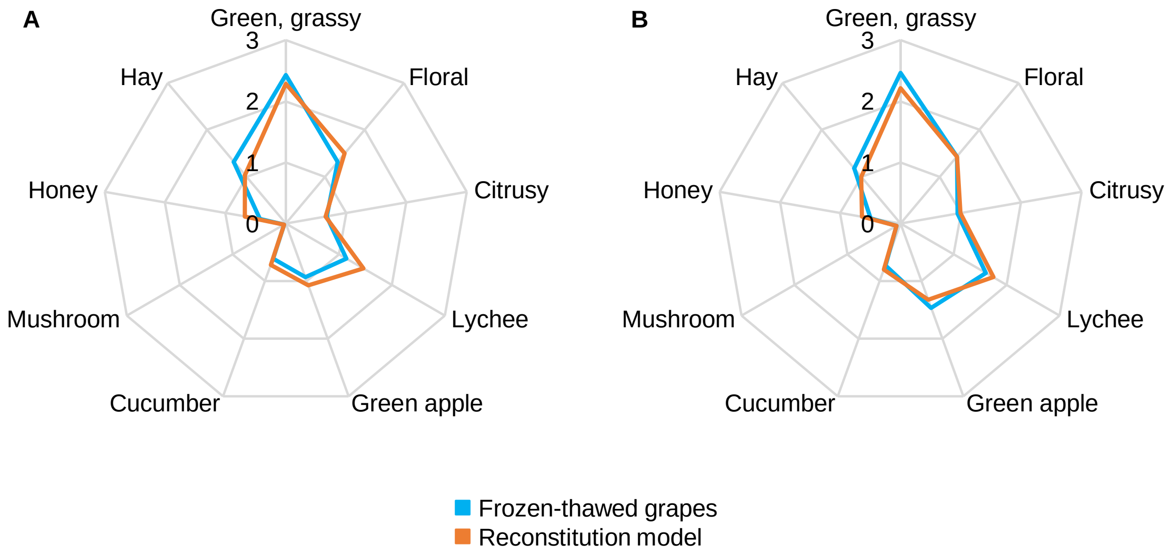

Figure S2. Quantitative olfactory profiles of the frozen-thawed Muscaris (A) and Muskateller (B) grapes in comparison to the quantitative olfactory profiles of the respective odor reconstitution models. Assessors rated the intensity of each descriptor on a scale from 0 to 3 with 0.5 increments and 0 = not detectable, 1 = weak, 2 = moderate, and 3 = strong.
